# Clinical outcomes of tocilizumab therapy in polyarticular and systemic juvenile idiopathic arthritis: a single-center analysis (2018-2022)

**DOI:** 10.1007/s00296-024-05711-4

**Published:** 2024-09-23

**Authors:** Agnieszka Gazda, Iryna Naishtetik, Beata Kołodziejczyk, Khrystyna Rybak, Małgorzata Mańczak, Joanna Wójtowicz, Olga Krasowicz-Towalska, Piotr Gietka

**Affiliations:** 1 Clinic and Polyclinic of Rheumatology of Developmental Age, National Institute of Geriatrics, Rheumatology and Rehabilitation in Warsaw, 1 Spartanska Street, Warsaw, 02-637 Poland; 2 Department of Gerontology, Public Health and Didactics, National Institute of Geriatrics, Rheumatology and Rehabilitation in Warsaw, 1 Spartanska Street, Warsaw, 02-637 Poland

**Keywords:** Juvenile Idiopathic Arthritis, Tocilizumab, safety and efficacy

## Abstract

This single-center retrospective study aimed to evaluate the safety and efficacy of Tocilizumab (TOC) in children with polyarticular (pJIA) and systemic juvenile idiopathic arthritis (sJIA) who exhibited inadequate responses to disease-modifying antirheumatic drugs (DMARDs) and biological modifiers (bDMARDs). Conducted at the Department of Pediatric Rheumatology, National Institute of Geriatrics, Rheumatology, and Rehabilitation in Warsaw, Poland, between 2018 and 2022, the study enrolled 29 patients diagnosed with JIA based on International League of Associations for Rheumatology (ILAR) criteria. The cohort comprised 13 sJIA and 16 pJIA patients, aged 2-18 years, receiving TOC treatment for 24 months. Safety and efficacy assessments included analysis of medical documentation, laboratory tests (CRP, ESR, WBC), and Juvenile Disease Activity Score (JADAS) 71 at baseline, 3, 6, 12, and 24 months post-treatment initiation. Significant reductions in CRP and ESR levels were observed within three months, with sustained improvement in JADAS71 scores over the 24-month treatment period. A substantial majority, 73.07% of patients, achieved inactive disease status or low disease activity, highlighting T0C’s effectiveness. Adverse effects were manageable, predominantly involving mild to moderate infections, with no serious adverse events or instances of macrophage activation syndrome (MAS). The study also noted a steroid-sparing effect of TOC, with a reduction in glucocorticoid usage among the cohort. Tocilizumab demonstrates substantial efficacy in reducing disease activity and improving clinical outcomes in patients with pJIA and sJIA, coupled with a favorable safety profile. These findings reinforce the role of TOC as a critical component of the therapeutic arsenal for JIA, offering hope for improved quality of life and disease management in this patient population.

**Supplementary Information:**

The online version contains supplementary material available at 10.1007/s00296-024-05711-4.

## Background

Juvenile idiopathic arthritis (JIA) represents a significant public health challenge as it is the most common form of arthritis in children, occurring before the age of 16 and lasting over six weeks [1, 2]. This condition, characterized by persistent joint inflammation, can lead to debilitating joint damage, hinder normal growth and result in substantial long-term disabilities, severely impacting the quality of life of affected individuals [3, 4]. Globally, JIA shows a prevalence of approximately 100 per 100,000 children, making it a notable concern in pediatric rheumatology [5]. Despite its prevalence, comprehensive epidemiological data, particularly in specific regions such as Poland, remains scarce. This lack of detailed epidemiological studies and patient registries hampers the understanding of the disease’s true impact, with only rough estimates available indicating a diagnosis rate of 2.6–10 per 100,000 children in the Polish pediatric population [6, 7].

The complexity of JIA is further evidenced by its classification into seven distinct subtypes as per the International League of Associations for Rheumatology (ILAR) criteria [1, 4]. In subtype polyarticular JIA (pJIA) with or without the presence of RF the disease onset is characterized by the involvement of five or more joints, and is known for its potential to cause profound disability, with up to 30% of patients continuing to exhibit active disease despite treatment with methotrexate or biologic agents [8]. Systemic JIA with clinical manifestations in addition to arthritis of systemic symptoms: fever, erythematosus rash, hepatosplenomegaly, lymphadenopathy, serositis, is less common but notorious for its severity and complications.The most serious complication of sJIA is macrophage activation syndrome (MAS), which contributes disproportionately to JIA-related morbidity and mortality [9–11]. The treatment of JIA is challenging [12, 13], particularly the treatment of pJIA and sJIA subtypes when there is an inadequate response to conventional therapies such as non-biologic disease-modifying antirheumatic drugs (DMARDs) [4, 14]. The chronic use of systemic glucocorticoids (GCS), a common component of JIA management, is fraught with risks including growth suppression, bone fractures, cataracts [15, 16].

The advent of biologic therapies targeting specific inflammatory mediators has revolutionized JIA treatment. These therapies, which include inhibitors of tumor necrosis factor (TNF), interleukin (IL)-1, and IL-6, and more recently IL17 inhibitors and janus kinase inhibitors, offer a more targeted approach, addressing the underlying inflammatory processes of the various JIA types [17–19].

Interleukin-6 (IL-6) is a proinflammatory cytokine that significantly contributes to both the articular and extra-articular manifestations of juvenile idiopathic arthritis (JIA), as well as to the chronic complications associated with the disease. Serum concentrations of IL-6 are correlated with the extent and severity of joint involvement, fever patterns, platelet counts, growth retardation and osteoporosis. Tocilizumab, a recombinant humanized monoclonal antibody, functions as an IL-6 receptor (IL-6R) antagonist. In 2011, tocilizumab was approved by the United States and Europe for the treatment of systemic JIA (sJIA) in children, and in 2013, it received approval for the treatment of polyarticular JIA (pJIA) [20–22].

Clinical trials and early treatments with TOC have demonstrated its profound and lasting impact on both systemic and articular symptoms of severe JIA, particularly in cases where the disease was refractory to other treatment modalities [14, 23–27]. The aim of the study was to determine the safety and efficacy of tocilizumab in patients with pJIA, sJIA, who had inadequate responses to DMARDs, bDMARDs in a single-centre.

**Fig. 1:**
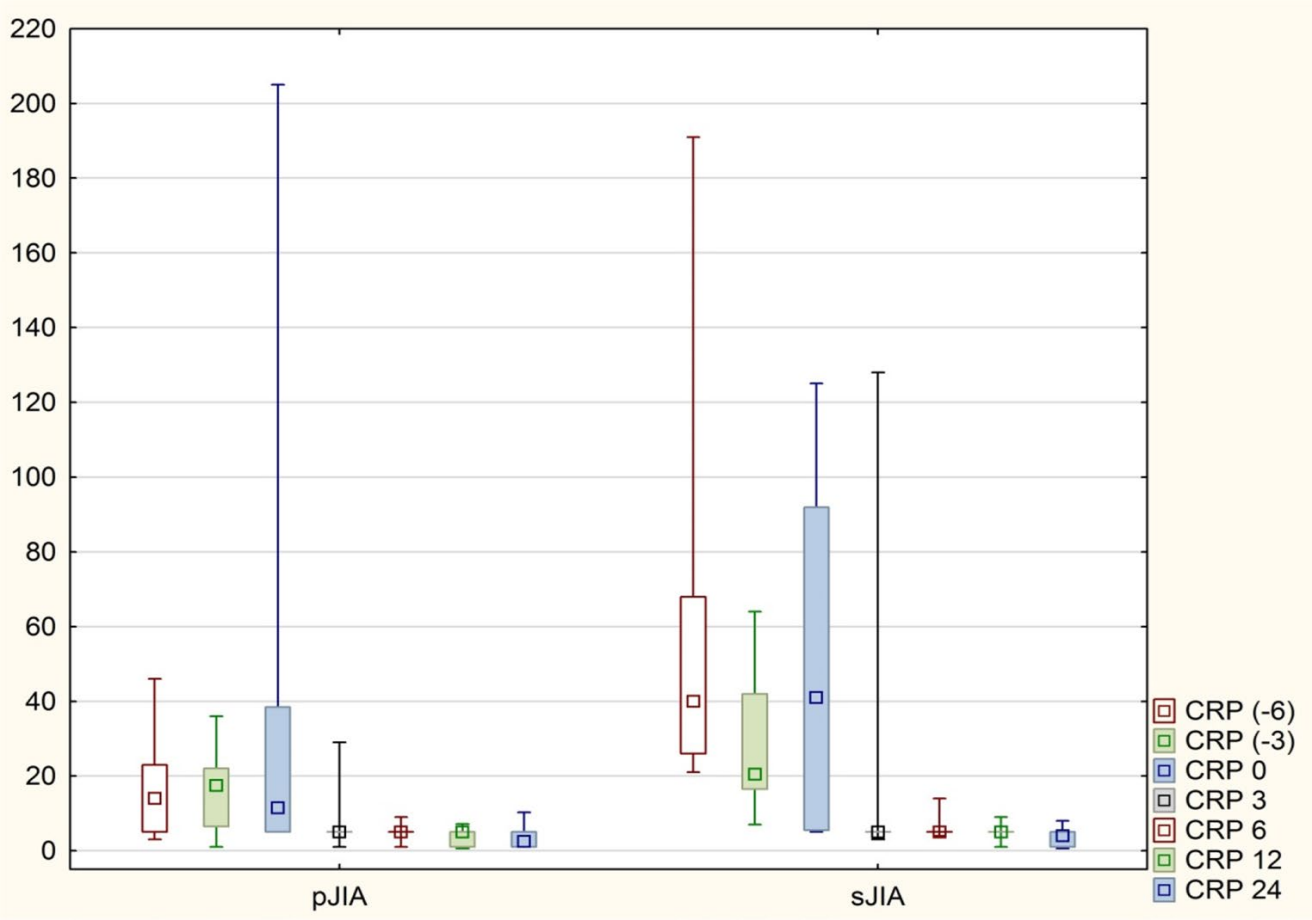
Levels of CRP in both groups before TOC and during treatment period

**Fig. 2:**
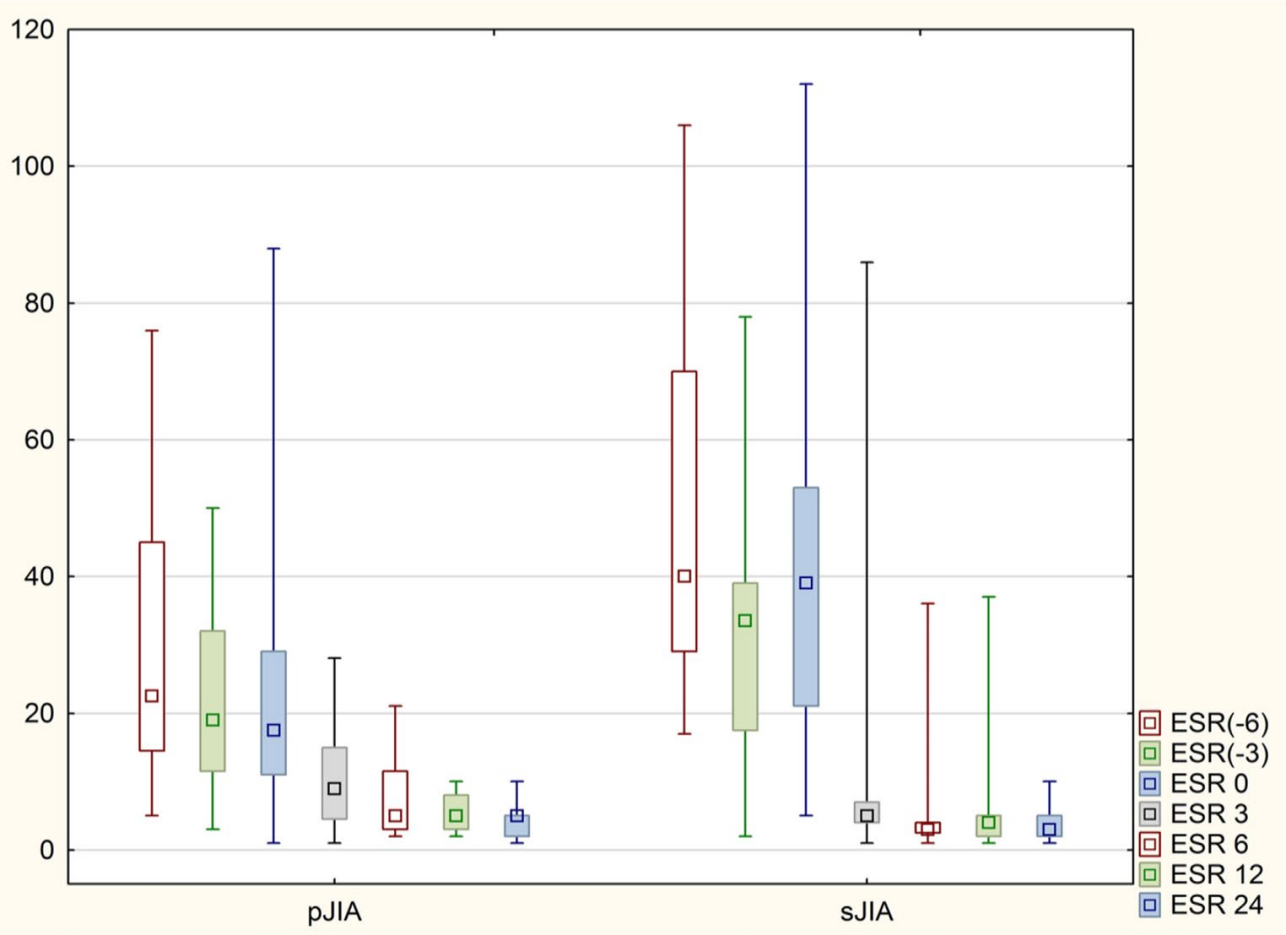
Levels of ESR in both groups before TOC and during treatment period

**Fig. 3:**
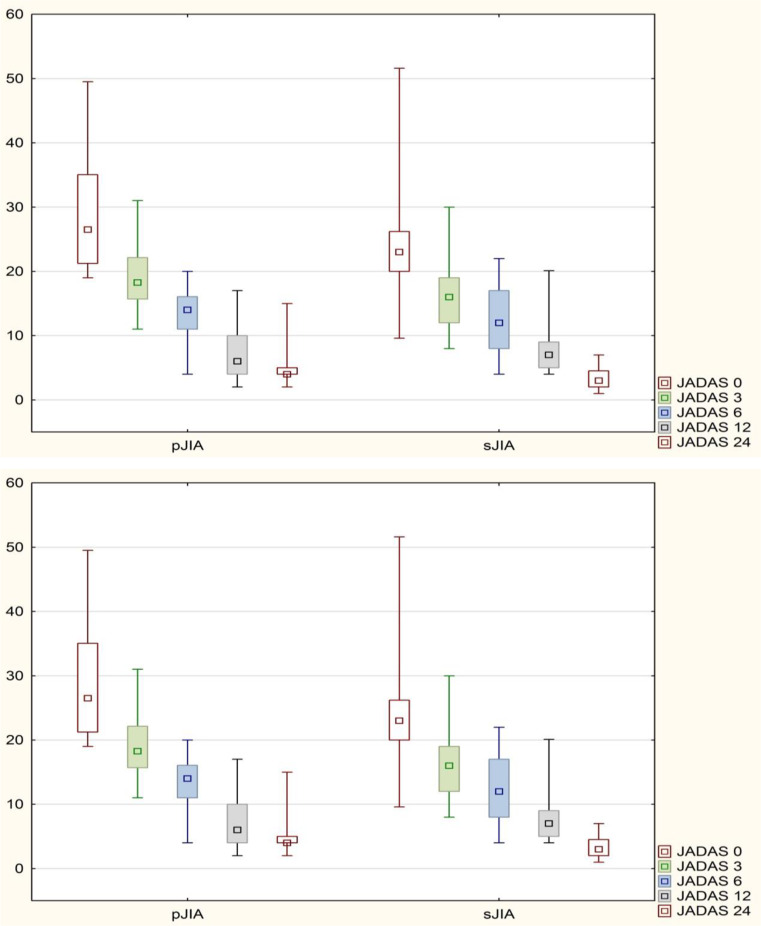
JADAS71 in both groups during treatment period

## Materials and methods

Single-center retrospective study were provided in the Clinic of Rheumatology of Developmental Age, National Institute of Geriatrics, Rheumatology and Rehabilitation in Warsaw Poland between 2018 and 2022.

Inclusion criteria necessitate a diagnosis of Juvenile Idiopathic Arthritis (JIA) based on the classification criteria established by the International League of Associations for Rheumatology (ILAR) for patients aged between 2 and 16 years [1]. Eligible patients must have been treated with Tocilizumab (TOC) for systemic JIA (sJIA) or polyarticular JIA (pJIA) within the study timeframe, with prescribed TOC dosages of 12 mg/kg/dose for sJIA patients weighing less than 30 kg and 8 mg/kg for those weighing more than 30 kg, administered biweekly, and 10 mg/kg for pJIA patients weighing less than 30 kg and 8 mg/kg for those weighing more than 30 kg, administered every four weeks. Patients are permitted to use NSAIDs, DMARDs, and glucocorticosteroids (GCS) at a dosage of less than 1 mg/kg/day, provided the dosage remained stable for a minimum of two weeks prior to the trial. Informed consent must be obtained from the parents or guardians of the participating children.

Exclusion criteria included the presence of other rheumatic diseases, infections, neoplastic conditions, surgical disorders or any other medical condition that could influence the studied parameters. including leukopenia (< 3.5 × 10^9/L) or thrombocytopenia (< 100 × 10^9/L).

The medical documentation was analyzed, including the assessment of subjective and objective examinations, results of laboratory tests, and imaging studies.

Laboratory examinations, which included laboratory blood tests: alanine aminotransferase (ALT), aspartate aminotransferase (AST), amount of white blood cell count (WBC), neutrophils, erythrocytes, hemoglobin (Hb), platelets, ESR, CRP, lipidogram were analyzed at baseline, after 3 months, 6 months, 12 months and 24 months.

Safety was assessed by reporting side effects as drug reactions, infections, levels of white blood cells, neutrophiles, thrombocytes, transaminases, lipidogram. To estimate side effects, all available laboratory results performed in pJIA every 4 weeks, in sJIA every 2 weeks were analyzed.

Leukopenia defined as leukocytes below 3 ˟10 9 L with mild leukopenia (range 3–4 ˟10 9 L); neutropenia (level of absolute neutrophil count < 1.5 ˟10 9 L), was classified as mild neutropenia- grade 2 (the range of neutrophils 1-1.5 × 10 9 /L), moderate neutropenia- grade 3 (neutrophils 0.5 to 1.0 × 10^9/L), severe neutropenia-grade 4 -(neutrophils < 0.5 × 10^9/L) [28].

Laboratory measurements were assessed according to Common Terminology Criteria for Adverse Events, version 3.0. Patients with elevations in liver enzymes were evaluated for hepatic injury [i.e., according to Hy’s law, with elevations of ALT or AST grade 2 defined as 3–5 times the upper limit of normal (ULN) grade 3 (5–20 times ULN) [29, 30].

Response to TOC was assessed in both groups by Juvenile Arthritis Disease Activity Score (JADAS) 71 at baseline and 3,6,12,24 months after initiation of the treatment. The JADAS-71 score specifically refers to the version of the JADAS that includes an active joint count up to 71 joints, making it comprehensive and thus suitable for all JIA subtypes. The score is calculated as follows: JADAS71 = Physician’s Global Assessment (0–10) + Parent/Patient Global Assessment (0–10) + Active Joint Count (0–71) + ESR(normalized 0–10) [31].

The result is a number that can range from 0 to above 100, with a higher score representing higher disease activity. Values of disease activity were divided into 4 classes according to polyarthritis JADAS activity: inactive (˂1), low disease activity (1.1–3.8), moderate (3.9–10.5) and high disease activity (˃10,5) [32]. Patients with inactive or low disease activity were considered as responders. Active systemic manifestations in patients with sJIA were included in Physician’s Global Assessment and Parent/Patient Global Assessment. The data about gender, age, clinical symptoms, adverse events, medications, which were used before treatment and during treatment period, were collected and statistically analysed.

The protocol and amendments were approved by the Institutional review board (approval number KBT- 4/3/2022), we obtained informed consents from parents whose children participated in our study.

### Statistical analysis

All collected data were analyzed using Statistica 13. Distribution of continuous variables was verified using the Shapiro-Wilk test. Normal distributed variablesare presented as mean and standard deviation (SD), not normal distributed variables as median and interquartile range (IQR). Nominal variables are presented as numbers and percentages. Mann–Whitney U test and χ2 test were used to compare baseline characteristics in both study groups (sJIA and pJIA). Differences between variables for different time points were analyzed using the Wilcoxon paired order test. Level of statistical significance was set at *p* < 0.05.

## Results

In this research study, we examined 29 children the baseline characteristics of two distinct patient groups: those with pJIA and sJIA. The sample included 16 patients in the pJIA group, with a gender distribution of 19% boys and 81% girls, and 13 patients in the sJIA group, comprising 15% boys and 85% girls. One patient from pJIA group had RF and anty-CCP anybodies. The mean age of diagnosis across both groups was similar pJIA- 5.4 (with a standard deviation of 2.4 years), sJIA 6.2 years (SD 4.0 years), and the youngest participant was nearly 5 years old. The average age at TOC initiation being 9.9 years (SD = 2.8) for the pJIA group and 8.6 years (SD = 3.3) for the sJIA group. Notably, the average time from diagnosis to TOC initiation differed between groups, being 3.9 years (SD = 0.8–7.7) for the pJIA group and 0.9 years (SD = 0.2–10.9) for the sJIA group.(Table 1).

**Table 1:** Baseline characteristics of patients, medications used before TOC, laboratory markers and JADAS at baseline

	pJIA n = 16	sJIA n = 13	p
Female	13 (81%)	11 (85%)	0.604
Age at diagnosis	5.4 (2.4)	6.2 (4.0)	0.779
Age at TOC initiation	9.9 (4.4)	8.6 (3.3)	0.502
Time from diagnosis to TOC initiation	3.9 (0.8–7.7)	0.9 (0.2–10.9)	0.055
bDMARDs (anti -TNFα inhibitors)	6 (38%)	3 (23%)	0.336
GCS	10 (67%)	11 (92%)	0.277
MTX	16 (100%)	11 (85%)	0.192
CsA	6 (40%)	8 (62%)	0.449
HCQ	12 (75%)	1 (8%)	**< 0.001**
SSZ	3 (20%)	0 (0%)	0.139
JADAS	26.5 (21.3 35.1)	23.0 (20.0 26.2)	0.215
Hb	12.4 (11.9 13.2)	11.7 (10.4 13.0)	0.133
CRP	11.5 (5.0–38.5)	41.0 (5.5 − 92.0)	0.241
ESR	17.5 (11.0 29.0)	39.0 (21.0 53.0)	**0.053**
PLT	339 (290–446)	381 (289–454)	0.698
WBC	8.7 (7.2–11.1)	14.3 (9.6 − 21.9)	**0.011**
NEUT %	56.0 (50.6–64.0)	66.3 (50.7 71.1)	0.223
NEUT	4.7 (3.8–6.7)	8.7 (6.3–14.5)	**0.008**
ALT	17.5 (15.5 27.0)	18.0 (11.5 34.0)	1
AST	32.0 (23.0 36.5)	25.5 (24.0 35.0)	0.698

All patients from both groups exhibited arthritis and demonstrated elevated disease activity as quantified by the JADAS71. The median JADAS71 score in the pJIA cohort was recorded at 26.5, with IQR from 21.3 to 35.1. Similarly, the sJIA cohort exhibited a pre-treatment median JADAS71 score of 23.0, with IQR from 20.0 to 26.2.

At the baseline only 5 patients from sJIA group presented fever, rash, most of the patients had a long history of treatment with DMARDS previously. Interestingly, 5 patients in the sJIA group developed Macrophage Activation Syndrome (MAS) during their disease course before initiation of treatment TOC.

Patients in both groups had previously been treated with DMARDs, GCS and some bDMARDs. When comparing disease activity between the pJIA and sJIA groups, we observed that the sJIA group exhibited higher ESR and WBC counts than the pJIA group at baseline. This difference was borderline significant for ESR (*p* = 0.053) and statistically significant for WBC (*p* = 0.011) and neutrophils count (*p* = 0.008) (Table 1).

We analyzed the laboratory parameters of patients in the period of 6 and 3 months prior to the initiation of TOC treatment, the average value of CRP in the pJIA group was 14 mg/l -6 months before TOC and 17.5 mg/l -3 months before TOC, in the sJIA group there were higher values of 40 mg/l and 20 mg/l respectively, ESR in the pJIA group 6 months before TOC was 22.5 mg/l and 3 months before 19 mg/l, in the sJIA group it was 40 mm/h and 33.5 mm/h respectively. There were no statistical differences in the values of ESR, CRP, leukocytes comparing the two groups in this period.

The exception was AST levels in the sJIA group. AST levels were notably lower 6 months (*p* = 0.015) and 3 months (*p* = 0.053, borderline significant) before the initiation of biological treatment, compared to the levels at the time of treatment commencement. No alterations in ALT levels were observed.

Patients within the systemic juvenile idiopathic arthritis (sJIA) group who exhibited systemic manifestations at the ontset of the study(5 patients) showed a remarkable absence of these systemic symptoms following the administration of the second dose of TOC.

Significant statistical changes were observed in CRP, ESR, Platelet count (PLT), and JADAS 71 as early as 3 months following the initiation of TOC treatment in both groups. These changes continued to be significant at 6, 12, and 24 months post-treatment initiation (Table 2, Figs. 1, 2, 3).

**Table 2:** Treatment of TOC

pJIA	baseline	3	p baseline vs 3	6	p baseline vs 6	12	p baseline vs 12	24	p baseline vs 24
JADAS	26.5 (21.3–35.1)	18.3 (15.7–22.2)	< 0.001	14.0 (11.0–16.1)	< 0.001	6.0 (4.0–102.0)	0.001	4.0 (4.0–5.0)	0.001
Hb	12.4 (11.9–13.2)	12.9 (12.5–13.8)	0.056	13.2 (12.3–13.5)	0.100	13.3 (12.3–13.7)	0.191	12.9 (12.0–13.7)	0.443
CRP	11.5 (5.0–38.5)	5 (5–5)	0.005	5 (5–5)	0.005	5 (1–5)	0.002	3 (1–5)	< 0.001
ESR	17.5 (11.0–29.0)	9 (5–15)	0.002	5 (3–12)	0.001	5 (3–8)	0.001	5 (2–5)	0.001
PLT	339 (290–446)	293 (250–336)	0.013	274 (242–346)	0.004	274 (248–306)	0.011	276 (250–365)	0.028
WBC	8.7 (7.2–11.1)	7.2 (5.8–8.4)	0.031	7.2 (5.1–8.0)	0.003	6.5 (5.1–8.1)	0.023	6.7 (5.6–8.7)	0.010
NEUT%	56.0 (50.6–64.0)	54.7 (45.8–64.7)	0.836	52.3 (43.6–66.5)	0.453	48.9 (45.3–56.4)	0.196	48.5 (43.5–63.1)	0.326
NEUT	4.7 (3.8–6.7)	3.9 (2.9–5.0)	0.079	3.3 (2.3–4.6)	0.006	3.0 (2.6–4.4)	0.039	3.1 (2.5–5.0)	0.020
ALT	17.5 (15.5–27.0)	24 (17–35)	0.198	23 (15–29)	0.670	18 (16–24)	0.410	15 (11–19)	0.140
AST	32.0 (23.0–36.5)	30 (23–37)	0.638	31 (26–36)	0.820	28 (25–36)	0.975	22 (17–27)	0.012
**sJIA**	baseline	3	p baseline vs 3	6	p baseline vs 6	12	p baseline vs 12	24	p baseline vs 24
JADAS	23.0 (20.0 26.2)	16.0 (12.0–19.0)	0.003	12.0 (8.0–17.0)	0.003	7.0 (5.0–9.0)	0.002	3.0 (5.0–4.5)	0.002
Hb	11.7 (10.4 13.0)	13.2 (12.0–14.0)	0.022	13.0 (11.7–14.0)	0.023	13.5 (12.3–14.1)	0.008	13.4 (12.8–13.9)	0.045
CRP	41.0 (5.5 − 92.0)	5 (5–5)	0.013	5 (5–5)	0.011	5 (5–5)	0.008	4 (1–5)	0.008
ESR	39.0 (21.0 53.0)	5 (4–7)	0.002	3 (3–4)	0.002	4 (2–5)	0.002	3 (2–5)	0.002
PLT	381 (289–454)	293 (268–321)	0.041	269 (232–300)	0.028	277 (263–343)	0.028	263 (242–325)	0.034
WBC	14.3 (9.6 − 21.9)	7.8 (6.7–8.8)	0.004	6.6 (5.7–8.4)	0.004	6.3 (4.9–7.0)	0.002	5.4 (4.7–6.6)	0.012
NEUT%	66.3 (50.7 71.1)	61.1 (44.9–67.4)	0.021	51.3 (37.7–59.5)	0.136	52.4 (42.5–57.1)	0.015	46.7 (37.0–58.5)	0.099
NEUT	8.7 (6.3–14.5)	4.2 (2.9–5.6)	0.005	3.3 (2.2–5.1)	0.006	3.3 (2.2–4.6)	0.005	2.6 (1.9–3.5)	0.019
ALT	18.0 (11.5 34.0)	22 (20–31)	0.209	25 (17–37)	0.477	20 (15–23)	0.969	22 (14–23)	0.824
AST	25.5 (24.0 35.0)	33 (25–37)	0.209	33 (31–38)	0.078	30 (25–35)	0.456	31 (24–35)	0.638

Before treatment, the median CRP levels were 11.5 mg/l in the pJIA group and 41 mg/l in the sJIA group. Post-treatment, these levels reduced to 3 mg/l and 4 mg/l, respectively. (Fig. 1) Similarly, median ESR levels decreased from 17.5 mm/h to 5 mm/h in the pJIA group and from 39 mm/h to 3 mm/h in the sJIA group after treatment. (Fig. 2) There were no statistically significant changes in CRP and ESR levels between 12 and 24 months of treatment. The median JADAS71 score at baseline was 26.5 for the pJIA group and 23 for the sJIA group; these scores significantly improved to 4 and 3, respectively, by the end of the trial.(Table 2, Fig. 3).

Initially, a predominant proportion of patients presented with high disease activity according to JADAS 71, accounting for 96.5%, while a smaller fraction exhibited moderate disease activity, representing 3.5%. Subsequent assessments revealed a notable shift in disease activity over time.

After 3 months of treatment high activity was still in 93.1%patients, average in 6.89%, but at 6 months high activity was found in 68.9% of patients, average in 17.2%, low activity/inactive disease in 10.3%. After a year, high activity is 17.8%, average 57%, low activity / inactive disease 25%. At the 24-month evaluation, a substantial 73.07% of patients achieved either inactive disease status or low disease activity. In contrast, 19.93% continued to display moderate disease activity, and 7% were categorized as having high disease activity according to JADAS71 due to joint involvement in both groups.

Hb values showed no significant changes in the pJIA group during treatment, whereas in the sJIA group, a significant change was observed, with none of the patients presenting anemia by the end of the TOC treatment period.

Furthermore, the AST value in the pJIA group 24 months after initiating treatment was statistically significantly lower than the baseline value. However, earlier assessments at 3, 6, and 12 months did not show a deviation from the baseline values.

At baseline, 72% (21 children) use GCS, predominantly in sJIA group. In the sJIA group, GCS was discontinued on average after 15 months for 34% of the patients (10 patients), while in the pJIA group, it was discontinued after an average of 9 months for 20% of the patients (6 patients). Notably, 17% of patients (5 patients) continued GCS treatment. It is important to highlight that the dosages of GCS in chronic treatment were maintained at minimal levels, specifically at 0.1 mg/kgmc/day.

### Adverse events

Throughout the study, a thorough monitoring of the patient cohort revealed no incidence of MAS. Additionally, no drug infusion-related skin reactions or anaphylactic responses were observed in any of the participants.

In the studied cohort, we observed a transient increase in transaminases in 24% (7 patients), of the latter grade 2 in 7% (2 sJIA patients), grade 3 in 17%- (5 patients – 4 p with sJIA,1pJIA),

Notably, a substantial elevation, of transaminases, predominantly in the sJIA group, was unrelated to MAS; except for one, these patients received concurrent MTX therapy. MTX discontinuation in two sJIA cases led to normalization of transaminase levels, and none of the patients necessitated cessation of TOC therapy due to hypertransaminasemia.

Leukopenia, WBC count below 3 × 10^9/L was a rare finding, observed in only 1 sJIA patient (3.4% of 29 patients), concurrent with herpes infection. Leukopenia, WBC counts between 3 and 4 × 10^9/L was more common, affecting 31% of patients (9 patients). Neutropenia, with counts below 1 × 10^9/L was documented in 17.2% of patients, while 24% had counts between 1 and 1.5 × 10^9/L These hematologic findings were transient, and treatment was not discontinued as a result.

Hyperlipidemia presented in isolated cases; one sJIA patient exhibited an initial elevation in cholesterol (247 mg/dL) and triglycerides (TG) (224 mg/dL), which peaked at 331 mg/dL for cholesterol during treatment but returned to normal after one year. Similarly, another patient showed an initial high TG level (218 mg/dL), which fluctuated but eventually normalized. Elevated cholesterol was singularly noted once in a different patient (217 mg/dL).

Regarding the infectious profile, the rate of infection was 2.4 per patient-year in the whole group, in sJIA the score was higher 2,9 patient-year, in pJIA it was 1,8 per patient-year.

During the first 6 months of treatment 51.7% of patients experienced upper respiratory tract infections, with 24.1% encountering recurrent episodes; 20.6% necessitated antibiotic treatment. Other infections included gastrointestinal infections in 6.8% of the cohort, herpes simplex virus type 1 (HSV-1) reactivation in 10.3%, stomatitis caused by Candida albicans in 6.8%, and solitary incidents of various viral and bacterial infections (Table 3).

**Table 3:** Infectious AE during TOC therapy

Infections	0-6 months	7–24 months
Upper respiratory tract infection	51,7%	79,3%
Bronchitis	3,4%	20,6%
Pneumonia		3,4%
	6,8%	6,8%
HSV-1 reactivation	10,3%	24,1%
Stomatitis caused by C.albicans	6,8%	
Urinary tract infection		3,4%
Acute otitis media		3,4%
Latent tuberculosis		3,4%
Chickenpox	3,4%	3,4%
Hand, Foot and Mouth Disease (HFMS)	3,4%	
Giardiasis		3,4%
Pinworm		3,4%
Soft tissue inflammation	3,4%	3,4%
Fever of unknown etiology	3,4%	3,4%

Over the subsequent 18 months, upper respiratory tract infections were noted in 79.3% of patients, with recurrent episodes in 55.1%; 31% required antibiotics. Two children were confirmed to have COVID-19 infection, presenting with mild upper respiratory tract symptoms, one was from the sJIA group and the other was from the pJIA group. Neither required hospitalization. Broader infectious complications included bronchitis in 20.6% (majority requiring antibiotics), pneumonia in 3.4%, urinary and gastrointestinal tract infections in 6.8%, and HSV-1 reactivation in 24.1% with repeat episodes in one patient previously affected. Additional infections encompassed a singular recurrent otitis media case. In one patient was diagnosis of latent tuberculosis, was managed with prophylactic treatment, no incidence of active tuberculosis. Other isolated incidences included fever of unknown origin, soft tissue inflammation, chickenpox, giardiasis and enteritis. None of the patients had infections that met the criteria for severe advers events(SAE) or severe infection; patients were not hospitalized.

## Discussion

The present study’s findings are consistent with the established efficacy of tocilizumab in reducing disease activity in juvenile idiopathic arthritis. The majority of studies reported improvements in disease activity, measured by the JADAS71 or the American College of Rheumatology (ACR) criteria, in JIA patients treated with TOC. Specifically, TOC was shown to improve involved joint *counts*, ESR, CRP levels, physician global assessment, patient global assessment, and pain in both polyarticular and systemic JIA patients [20, 21, 24, 25]. Our results demonstrated a prompt and sustained response to TOC in both pJIA and sJIA subtypes, as evidenced by improved JADAS71 scores and laboratory markers (CRP, ESR) within 3 months of initiation. We observed considerable improvement according to JADAS71 score during 24 months. At the two-year mark of the study, a significant proportion of patients, amounting to 73.07%, reached a state of either inactive disease or demonstrated low disease activity. Conversely, 19.93% of the patients persisted in exhibiting moderate disease activity, while 7% were classified as experiencing high disease activity.

In Horneff et al. observation in 74 patients with mean treatment duration 0.98 ± 0.59 year in TOC cohort, the improvement at 24 months, JADAS minimal disease activity was achieved in 52.4% and JADAS remission in 27.9% of patients [22].

The international CHERISH trial has shown that extended treatment with TOC for a duration of 104 to 131 weeks or more is both safe and effective for managing pJIA. It was observed that the number of pJIA patients achieving inactive disease status rose from 26 patients (representing 63.4%) at baseline to 31 patients (equating to 75.6%) by visit 7, which corresponds to week 24 of the study [25, 26].

The international TENDER study enrolled 112 children aged between 2 and 17 years, all diagnosed with active sJIA. At the conclusion of week 52, 80% of the participants treated with TOC exhibited a minimum of 70% improvement in symptoms without any fever. Notably, within this group, 59% demonstrated a 90% symptom improvement. Furthermore, 48% of these patients reached a state where no joints exhibited active arthritis [24].

This rapid onset of action aligns with studies by Mohammed A Muzaffer et al. [33], which also reported early response in inflammatory markers.

Further, the notable reduction in GCS usage without loss of disease control adds to the growing body of evidence supporting TOC’s steroid-sparing benefits [34]. De Benedetti and colleagues observed that by the conclusion of the study, 52% of the participants had ceased using oral GCS [24]. Significantly, by the conclusion of the 24-week period, in our study 17% of the patients (comprising 5 patients) remained on GCS therapy. It is essential to emphasize that the dosages of GCS administered for chronic treatment were kept at minimal levels, specifically at a dosage of 0.1 mg/kg mc/day.This is an especially relevant finding considering the long-term side effects of steroids in pediatric populations.

Interestingly, in our sJIA cohort, the increase in AST prior to tocilizumab administration does not conform with current literature, which may indicate a long-term treatment DMARDs.

In this research, the safety profile of TOC, as observed in our cohort, aligns with existing literature, demonstrating a manageable spectrum of adverse effects. The most frequent adverse event, infections, was predominantly of mild to moderate severity in our cohort, with an infection rate of 2.34 infections per patient-year, with the higher incidence in sJIA group- score 2,9 patient-year, in pJIA it was 1.8 per patient-year.

These primarily included common childhood viral infections of the upper respiratory tract, bronchitis, and in one case, outpatient-treated pneumonia.

Comparatively, in the double-blind phase of the TENDER trial, the rate of infection per patient-year was higher in the tocilizumab group (3.4) compared to the placebo group (2.9), with the rate of severe infection being relatively low (0.115/patient-year at 52 weeks and 0.109 at 104 weeks) [24].

Infection rates in the group 71 sJIA patients receiving TOC according to German Biologics register (BiKeR) were significantly higher (RR 11.0 patient-year), while serious infections were rare in 2 children [35].

In the longitudinal data from the phase III Japanese study of tocilizumab in sJIA by Yokota et al., the rate of serious infection was at 0.132 events per person-year [13]. In the Japan registry in the group of 417 sJIA patients on tocilizumab rate of serious infection was 0.182 events per person-year [36].

In first meta-analysis of a randomized controlled trial to compare both the efficacy and safety of biological agents in patients with sJIA tocilizumab reported a statistically significantly increased risk of infections compared with placebo, but when evaluated as events per total patient-days, however, the risk was not increased [37].

Leukopenia (< 3 × 10^9/L) was rare, observed only once in a patient with sJIA (3.4% of the total 29 patients). Moderate neutropenia classified as grade 3 was observed in 17.2% of patients, with the lowest neutrophil count being 0.59 × 10^9/L. Yokota et al. reported grade 3 neutropenia in 17.9% of the patients [13]. Pardeo analyzed data for up to 2 years of TOC treatment in the TENDER and Cheerish trials, grade ≥ 3 neutropenia was observed in 25.0% of patients with sJIA and in 5.9% of patients with pcJIA [38].

Elevation of transaminases, in patients on TOC, was observed in our cohort, grade 2 were observed in 10% of sJIA patients, and significant increases (grade 3) occurred in 13% of patients. Elevation of transaminases in the TENDER trial occurred slightly less frequently- Grade 3 occurred in 6% of patients [24], in the trial by Yokota et al. i Grade 3 elevation of ALT and AST occurred in 9% and 6% of patients [13].

Alterations in lipid profiles were infrequent; in one patient in our study (3.5%), we observed an increase in cholesterol levels to a maximum of 1.50 times the norm. The TENDER trial reported similar low incidences of elevated total cholesterol and LDL levels.

Anaphylactic reactions, a concern with intravenous administration of TOC, did not occur in any of our patients. Literature review reveals MAS as a notable SAE, with several cases reported in other studies.

SAEs according to the literature were relatively rare, in TENDER trial it was 0.23 per PY in the open-label extension phase 0.25 at 52 weeks and 0.23 at 104 weeks; in the CHERISH trial the rate of SAEs was lower 0.093 per PY in the tocilizumab group and 0.109 per PY in the placebo group [24, 25]. According to the German Biologics register (BiKeR) in the group sJIA patients SAE were observed in TOC treatment sJIA patient with the RR 2.5 [35]. Literature review reveals MAS as a notable SAE, MAS on tocilizumab was reported in Yokota et al. study 2 patients, in the TENDER trial 3 patients, Shimizu et al. Describes 5 cases, in Japanese sJIA surveillance registry, the rate of MAS occurring in patients on tocilizumab (found to be 3.6%) was not determined to be higher when compared to the general sJIA population [13, 24, 39]. No SAEs were reported in our observation, none of our patients developed MAS during the 24 m-ths TOC treatment.

Our study was conducted in the context of the COVID-19 pandemic. All patients underwent COVID-19 testing before drug administration. None of the children developed PIMS. Two children were confirmed to have COVID-19 infection, presenting with mild upper respiratory tract symptoms. These children were family members of adults suspected of having COVID-19; one was from the sJIA group and the other was from the pJIA group. No patients discontinued their ongoing treatment or required hospitalization. However, these two pediatric patients postponed medication administration due to mild respiratory symptoms. According to the literature, unlike adults, children with JIA and COVID-19 tend to experience a milder clinical course, and asymptomatic infections are common [40–42].

Our study’s limitations include its single-center and retrospective nature, as well as the relatively small sample size without control group.

## Conclusions

The study conclusively demonstrates that TOC effectively reduces disease activity and improves clinical outcomes in patients with juvenile idiopathic arthritis, with notable advancements in both pJIA and sJIA subtypes. This effectiveness is underscored by significant improvements in JADAS71 scores and a marked reduction in systemic inflammation markers, positioning TOC as a pivotal treatment option for managing JIA.

Our study supports the existing evidence of a tolerable safety profile for TOC in the treatment of JIA, even during the COVID-19 pandemic. While adverse effects such as transient elevation of transaminases, leukopenia, and neutropenia are not uncommon, they are generally manageable and do not typically necessitate discontinuation of therapy. Future research should focus on long-term safety data and the optimization of treatment regimens to enhance patient outcomes.

## Electronic supplementary material

Below is the link to the electronic supplementary material.

Supplementary Material 1

Supplementary Material 2

Supplementary Material 3

Supplementary Material 4

Supplementary Material 5

Supplementary Material 6

Supplementary Material 7

Supplementary Material 8

## Data Availability

No datasets were generated or analysed during the current study.
